# Non-steroidal anti-inflammatory drug-induced anaphylaxis infrequent in 388 patients with mastocytosis: A two-center retrospective cohort study

**DOI:** 10.3389/falgy.2022.1071807

**Published:** 2022-12-05

**Authors:** Patrizia Bonadonna, Francesco Olivieri, Jesper Jarkvist, Francesca Nalin, Roberta Zanotti, Laura Maclachlan, Theo Gülen

**Affiliations:** ^1^ Allergy Unit, Department of Medicine, Azienda Ospedaliera Universitaria Integrata di Verona, Verona, Italy; ^2^ Department of Respiratory Medicine and Allergy, Karolinska University Hospital Huddinge, Stockholm, Sweden; ^3^ Hematology Unit, Department of Medicine, Azienda Ospedaliera Universitaria Integrata di Verona, Verona, Italy; ^4^ Institute of Environmental Medicine, Karolinska Institutet, Stockholm, Sweden; ^5^ Immunology and Allergy Unit, Department of Medicine Solna, Karolinska Institutet, Stockholm, Sweden; ^6^ Department of Medicine Huddinge, Karolinska Institutet, Stockholm, Sweden

**Keywords:** mastocytosis, NSAID (non-steroidal anti-inflammatory drug), anaphyalaxis, hypersensitivity, tryptase, igE, D816V KIT mutation

## Abstract

**Background:**

Anaphylaxis is a well-known feature of mastocytosis, particularly in relation to hymenoptera venom stings. It is therefore hypothesized that mastocytosis patients may also be predisposed to severe hypersensitivity reactions to certain medications including non-steroidal anti-inflammatory drugs (NSAIDs). For this reason, these patients are usually discouraged from using these drugs. The current study aimed to determine the prevalence and evaluate the severity of NSAID-related hypersensitivity reactions among patients with mastocytosis.

**Methods:**

A retrospective study was conducted among a total of 388 (≥18 years old) consecutive patients from two independent European mastocytosis centers, in Sweden and Italy. Patients underwent a thorough allergy work-up where self-reported NSAID-hypersensitivity reactions were re-evaluated by an allergist in the first cohort (202 patients) and results were validated in the second cohort (186 patients).

**Results:**

Overall frequency of NSAID-hypersensitivity was 11.3% in the total study cohort. Most patients reacted with cutaneous symptoms (89%), whereas severe hypersensitivity reactions were infrequent with only 11 patients (2.8%) experiencing anaphylaxis. All NSAID-related hypersensitivity reactions had occurred before mastocytosis was diagnosed. There was no difference between the groups regarding gender, baseline tryptase levels or presence of atopy, asthma/rhinitis.

**Conclusion:**

Our study indicates an approximate 4-fold increased prevalence of NSAID hypersensitivity among mastocytosis patients compared to the general population. However, most NSAID reactions were limited to the skin as the prevalence of overall anaphylaxis was infrequent. Our results support that mastocytosis patients with a known tolerance to NSAIDs can continue using these medications without special precautions, whereas those with a prior reaction to NSAIDs should undergo thorough allergy work-up, including drug challenges.

## Introduction

Mastocytosis encompasses a heterogeneous group of rare disorders characterized by the accumulation and activation of immunologically aberrant clonal mast cells (MCs) in various tissues and organs including the skin, bone marrow (BM) and the gastrointestinal (GI) tract (1–3). Mastocytosis can be systemic (SM) or cutaneous (CM) and in patients with monoclonal MC activation syndrome (MMAS), the World Health Organization (WHO) criteria for SM are not fully met (2, 3). These conditions are also referred to as clonal mast cell disorders (MCD) (1). In patients with mastocytosis, most probably, due to their inherent MC hyperreactivity, MC releasability may be increased (4) and, therefore, anaphylactic reactions are a well-known clinical feature of these patients (5–8). Hymenoptera venoms constitute the most common cause of anaphylaxis in mastocytosis, followed by unprovoked, i.e., idiopathic, anaphylaxis (7–10). However, the number of studies focusing on the association between drug hypersensitivity and mastocytosis is scarce (11–14). In one of these reports, authors evaluated different anesthetic techniques, such as general, epidural, and local anesthesia and sedation, in 501 mastocytosis patients by reviewing 676 procedures and reported an increased frequency of perioperative anaphylaxis in adult mastocytosis patients who previously presented with anaphylaxis (13). In contrast, although anaphylactic reactions were expected to be more frequent in patients with mastocytosis, this has not thus far been the case with reactions to COVID-19 vaccines (15, 16).

Drug hypersensitivity reactions (DHRs) to non-steroidal anti-inflammatory drugs (NSAIDs) are the second most common cause of DHRs, after antibiotics, representing an important health problem (17, 18). A US study reported an incidence of NSAID-induced anaphylaxis of 13 per 10,000 patients among 1.8 million patients seeking health care in the Greater Boston area (19). Moreover, the reported prevalence of DHR to NSAIDs ranges from 0.6 to 2.5% in the general population and reportedly higher among females and patients with chronic urticaria or asthma (20–23). It is hypothesized that, patients with mastocytosis may be predisposed to a higher risk of severe hypersensitivity reactions to NSAIDs. Nevertheless, very few studies have systematically investigated the prevalence and clinical features of NSAID hypersensitivity among patients with mastocytosis (24–27). The frequency of NSAID hypersensitivity in mastocytosis patients ranged from 2 to 14% (24–27). Additionally, patients with NSAID-induced anaphylaxis have been reported in the literature. In one case report, IgE-mediated metamizole anaphylaxis was confirmed in a patient with mastocytosis (28). In another study, two cases of anaphylaxis were reported after diclofenac ingestion in a cohort of 84 patients with SM (7). Another study addressed the question from a different perspective and evaluated whether there is a correlation between severe hypersensitivity reactions to NSAIDs and elevated serum baseline tryptase (sBT) levels among 286 patients with a history of NSAID-hypersensitivity (29). The authors were unable to demonstrate such a relationship, as only three patients had a diagnosis of SM (29).

Hence, there is an unmet need for further studies to determine the safety of NSAIDs in patients with mastocytosis, as it is not currently possible to provide clear recommendations. In the current study, we investigated the prevalence and clinical characteristics of DHR to NSAIDs in a large cohort of mastocytosis patients and validated these findings in an independent cohort of mastocytosis patients. Furthermore, we analyzed whether the occurrence and severity of reactions is influenced by certain risk factors, such as sBT levels, IgE levels, atopic status, gender or phenotype of MC disease.

## Materials and methods

### Study design and setting

A two-center retrospective cohort study was conducted among mastocytosis patients diagnosed and treated at the Mastocytosis Center Karolinska, Stockholm, Sweden, and the Verona Mastocytosis Center, Verona, Italy. A total of 388 adult mastocytosis patients were enrolled in the study.

The diagnosis of mastocytosis was obtained, in accordance with WHO-criteria (2, 3). Diagnostic workup included histopathological evaluation of bone marrow (BM), flow cytometry, *KIT* D816V mutation analysis, and measurement of sBT levels. Further, patient-reported NSAID reactions were reevaluated by allergists before considering them hypersensitivity reactions to NSAIDs. Patients with isolated gastrointestinal symptoms or vague complaints were excluded. A more detailed review of the medical records was performed for individuals with possible NSAID-induced anaphylaxis by two experienced allergists (*P*.B. and T.G.) to ensure that anaphylaxis diagnosis was supported by clinical findings and fulfilled the current criteria for anaphylaxis (30, 31). Atopy was defined as a positive skin prick test (SPT) result to at least one of the usually tested aeroallergens or a positive test to the inhalant, Phadiatop® (ImmunoCAP®, Thermo Fisher, Uppsala, Sweden).

### Study subjects

#### The Swedish cohort

The Mastocytosis Center Karolinska was established in 2006 at Karolinska University Hospital in Stockholm, Sweden, and receives referrals from the whole country. As of December 31st, 2018, 387 consecutive adult patients (>18 years) had been referred to the center due to suspected MC disorders. According to WHO-criteria, 186 patients met the criteria for the diagnosis of SM and additional 12 patients obtained diagnosis of mastocytosis in the skin (MIS) as they refused to undergo a BM-investigation. A further 33 patients were diagnosed with MMAS as they only fulfilled 1 or 2 criteria for SM. Of those 231 patients, we conducted a retrospective study among 202 patients, who provided their written consent and enrolled in the study. Ethical approval was obtained from the Regional Ethical Review Board, Stockholm, Sweden (approval no. 2011/1750-31/3 and 2018/2621-31).

All enrolled patients underwent standardized allergy work-up at the Respiratory Medicine and Allergy outpatient clinic, including detailed medical history and allergy tests, e.g., SPT and/or specific IgE antibody test (ImmunoCAP®), to assess the presence/absence of atopy, as previously described (3). Total IgE levels were measured in all patients, always together with sBT. In addition, detailed clinical information regarding demographic data, atopic status, history of previous reactions to NSAID, administration type, time between drug intake and symptom occurrence (when available), clinical manifestations, and clinical outcomes were obtained. The possible effect of general triggers, such as physical exertion, heat, cold, friction, emotional stress, alcohol, or histamine-containing food, was carefully evaluated. The likelihood of a possible NSAID-hypersensitivity was retrospectively assessed in individual patients by an experienced allergist (TG) by reviewing data from allergy work-up. Moreover, the status of patients has been updated during follow-up visits, and if a given patient subsequently developed a reaction, this information was also available. SPT was not performed with NSAIDs.

#### The Italian cohort

We obtained data from 186 patients with mastocytosis as per WHO criteria from the Verona Mastocytosis Center, “Gruppo Interdisciplinare per lo studio della Mastocitosi (GISM)”, as an independent validation cohort. The study was approved by the institutional review board of Verona University Hospital (protocol n° 1828 approved on 5 December 2010) and all enrolled patients gave their informed consent. All subjects underwent BM biopsies to obtain diagnosis. An allergy work-up to assess the presence/absence of atopy was performed; however, analysis of total IgE levels was principally performed only in patients who had a positive history of NSAID reactions. Among patients with reported-NSAID reactions, the likelihood of NSAID-hypersensitivity was assessed by an experienced allergist (PB). No SPT was performed with NSAIDs.

### Drug provocation test

An oral provocation test to NSAIDs was performed in 34 patients. Other patients declined the test due to anxiety about the outcome. Across the whole Italian cohort, 32 patients underwent a drug provocation test (DPT) using nimesulide, a non-selective NSAID which is licensed for use in certain countries. Of these 32, 24 were subjected to this procedure without any history of previous NSAID-hypersensitivity reactions, but they were reluctant to use NSAIDs due to widespread beliefs in the lay press. The remaining eight patients had previously reacted to some NSAIDs. Of those, five patients had a grade 1 reaction with acetylsalicylic acid (ASA), one patient had a grade 1 reaction with naproxen and another patient experienced a grade 1 reaction with ketoprofen. One patient had a grade 3 reaction with diclofenac. Furthermore, two patients in the Swedish cohort underwent DPT with ibuprofen, as both previously reacted with grade 1 reaction with this drug. All grades were reported as per the Ring & Messmer score (32). Thus, there was only one patient with a history of anaphylaxis among those 10 patients who underwent an NSAID challenge.

### Statistics

Statistical analyses were performed using SPSS 24.0 for Windows (SPSS Inc., Chicago, IL, USA), and a *p*-value of <0.05 was considered statistically significant. Median values and ranges are presented to describe continuous variables and frequencies for categorical variables. Because the distribution of these data was not normal, group differences were analyzed using the Mann–Whitney U-test. Categorical variables were analyzed with the Chi-Square test or Fisher's exact test, when appropriate.

## Results

### Patient demographic and characteristics

General patient characteristics are shown in Table 1. The largest group of patients in both cohorts were those with a diagnosis of SM, most had indolent SM (93% in the Swedish cohort and 95.6% in the Italian). Patients with skin engagement of mastocytosis constituted 62.4% of the SM group in the Swedish cohort, whereas only 37.1% in the Italian cohort (*p* < 0.01). Moreover, a minority of patients were diagnosed with MMAS (33 and 22, respectively) and MIS (12 and 5, respectively) (Table 1). Gender was relatively evenly represented in both cohorts (46.1% male in the Swedish cohort, 51.1% in the Italian). Median age at diagnosis was similar across both cohorts, 52 years in the Swedish cohort and 50 years in the Italian. Similarly, the median age of diagnosis was lower in patients with MIS than other phenotypes in both cohorts (37 and 42, respectively) (Table 1). Moreover, median follow-up times were 7 years (range 0.5–15 years) and 8 years (range 1–22 years) for the Swedish and Italian cohorts respectively.

**Table 1:** Demographics and baseline characteristics of patients enrolled to the study.

	Swedish cohort	Italian cohor
**Characteristics**	SM (*n* = 157)	MIS (*n* = 12)	MMAS (*n* = 33)	Total (*n* = 202)	SM (*n* = 159)	MIS (*n* = 5)	MMAS (n = 22)	Total (*n* = 186)
**Male gender, *n* (%)**	72 (45.9)	5 (41.7)	16 (48.5)	93 (46.1)	82 (51.6)	1 (20.0)	12 (54.5)	95 (51.1)
**Age at diagnosis, median (range)**	52 (18–84)	37.5 (18–76)	54 (28–74)	52 (18 –84)	50 (21-83)	42 (20-65)	48 (21-65)	50 (20-83)
**Presence of MC aggregates, *n* (%)**	87 (55.4) (3 n/a)	Not done	0 (0)	87 (43-0) (15 n/a)	61 (38.4) (25 n/a)	Not done	0 (0)	61 (32.8) (25 n/a)
**Presence of atypical morphology, *n* (%)**	147 (93.6) (4 n/a)	Not done	7 (22.5) (2 n/a)	154 (76.2) (17 n/a)	140 (88.1) (11 n/a)	Not done	8 (36.4) (9 n/a)	134 (72.0) (20 n/a)
**Presence of CD25, *n* (%)**	153 (97.5) (4 n/a)	Not done	27 (81.8) (1 n/a)	181 (89.6) (16 n/a)	142 (89.3) (14 n/a)	Not done	11 (50.0) (7 n/a)	148 (79.6) (21 n/a)
**^a^** **Presence of *KIT* D816V mutation, *n* (%)**	116 (83.5)	6 (50.0)	8 (24.2)	130 (71.4)	146 (91.8)	0	13 (59.1)	159 (85.5)
**Basal tryptase (ng/mL), median (range)**	30 (6–650)	12 (3–160)	9.4 (3–23)	24.5 (3–650)	20.9 (3,6–505)	13.5 (7–18.5)	14 (3.4–108)	20.2 (7–505)
**Presence of atopy, *n* (%)**	43 (27.4) (1 n/a)	4 (33.3) (1 n/a)	11 (33.3) (1 n/a)	58 (28.7) (3 n/a)	53 (33.3)	2 (40.0)	7 (31.8)	62 (33.3)
**Total IgE (kU/L), median (range)**	14 (1–1600)	31 (2–110)	35 (6–1100)	16 (1–1600)	31.5 (4-892)	(5 n/a)	33 (8.5–50)	31.5^b^ (4–892)
**History of any anaphylaxis, *n (%)***	78 (49.7)	0 (0.0)	23 (69.7)	101 (50.0)	74 (46.5)	2 (40.0)	12 (54.5)	88 47.3)

Abbreviations: SM, systemic mastocytosis; MIS, mastocytosis in the skin; MMAS, monoclonal mast cell activation syndrome; n/a = not applicable.

^a^ D816V mutation analysed in 182 patients in the Swedish cohort.

^b^ Total IgE test was performed only in 52 patients in the Italian cohort.

Regarding bone-marrow findings, the presence of the major criterion, i.e., the MC aggregates, was present in 55.4% of SM patients in the Swedish cohort and in 38.4% of the Italian. Furthermore, atypical morphology, presence of aberrant CD25 expression and presence of *KIT* D816V mutation was similar in both cohorts. Median baseline tryptase (ng/mL) for the whole Swedish cohort was 24.5 (range 3–650) and 20.2 (range 7–505) for the Italian. Levels were highest in the SM group in both cohorts (30 and 20.9, respectively).

Atopy was present in 28.7% of the Swedish cohort and 33.3% of the Italian cohort. Median total IgE (kU/L) was lower in the Swedish cohort (16 with a wider range 1–1,600), compared to the Italian cohort, 31.5 (4–892). However, in the Italian cohort, IgE levels were determined only in 52 patients, of whom 43 had reported hypersensitivity to NSAIDs. A history of anaphylaxis was similar across cohorts (50.0% in the Swedish cohort, 47.3% in the Italian).

### Prevalence of NSAID hypersensitivity

Of the 23 patients with self-reported NSAID hypersensitivity in the Swedish cohort, a likely hypersensitivity reaction was confirmed in 18 patients (8.9% of the total cohort) by the allergist after clinical re-assessment. We observed a 2-fold increased frequency of hypersensitivity among patients with MMAS (15.2%) compared to patients with mastocytosis (7.6%), however, the difference was not statistically significant (Table 2). In the Italian validation cohort, of the 43 patients with self-reported NSAID hypersensitivity, 26 patients (14%) were deemed to have NSAID hypersensitivity, after re-evaluation by the allergist. There was an increased frequency of NSAID hypersensitivity in the MMAS group (27.3%) compared to the mastocytosis group (12.2%) without reaching statistical significance. However, when we looked at the total study cohort, the frequency of NSAID hypersensitivity among patients with MMAS was significantly higher compared to mastocytosis patients (*p* = 0.038). NSAID-induced anaphylaxis was confirmed in 2.0% of the Swedish cohort and 3.8% of the Italian cohort. The frequency of NSAID-induced anaphylaxis was significantly higher among MMAS patients compared to mastocytosis patients (*p* = 0.037) in the Italian cohort, but not in the Swedish or the total study cohort (Table 2).

**Table 2:** Prevalence of NSAID-induced hypersensitivity reactions in the study subjects.

** **	** **	**Total**	**Mastocytosis**	**MMAS**	** **
**Swedish cohort**	** **	**(*n* = 202)**	**(*n* = 169)**	** (*n* = 33)**	**^a^** **p-value**
	**NSAID-induced hypersensitivity, *n* (%)**	**18 (8.9%)**	**13 (7.7%)**	**5 (15.2%)**	**0** **.** **18**
	**NSAID-induced anaphylaxis, *n* (%)**	**4 (2.0%)**	**3 (1.8%)**	**1 (3.0%)**	**0** **.** **51**
**Italian cohort**	** **	**(*n* = 186)**	**(*n* = 164)**	**(*n* = 22)**	** **
	**NSAID-induced hypersensitivity, *n* (%)**	**26 (14.0%)**	**20 (12.2%)**	**6 (27.3%)**	**0** **.** **09**
	**NSAID-induced anaphylaxis, *n* (%)**	**7 (3.8)**	**4 (2.4)**	**3 (13.6)**	**0** **.** **037**
**Total study cohort**	** **	**(*n* = 388)**	**(*n* = 333)**	**(*n* = 55)**	** **
	**NSAID-induced hypersensitivity, *n* (%)**	**44/388 (11.3%)**	**33/333 (9.9%)**	**11/55 (20.0%)**	**0** **.** **038**
	**NSAID-induced anaphylaxis, *n* (%)**	**11/388 (2.8%)**	**7/333 (2.1%)**	**4/55 (7.2)**	**0** **.** **055**

NSAID, non-steroidal anti-inflammatory drug; MMAS, monoclonal mast cell activation syndrome.

^a^ *p*-values were calculated using Chi-square test or Fisher's exact test.

### Clinical features of the reactions

**Figure 1:**
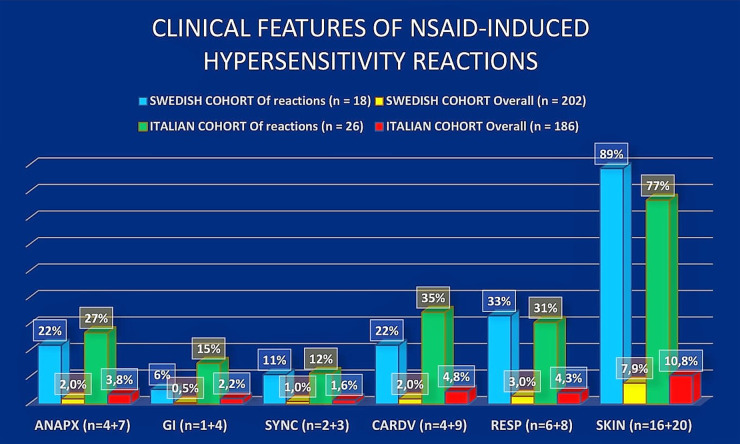
Figure demonstrates reaction pattern of NSAID-induced hypersensitivity reactions in Swedish and Italian cohorts. Abbreviations: ANAPX, anaphylaxis; SYNC, syncope; CARDV, cardiovascular; RESP, respiratory; GI, gastrointestinal.

The clinical patterns of NSAID reactions are shown in Figure 1. All NSAID-related hypersensitivity reactions occurred before mastocytosis was diagnosed. The reactions were mostly mild and limited to the skin; however, some anaphylactic reactions were also reported. There were no new onset NSAID reactions during post-diagnostic follow-up years.

Cutaneous symptoms were the most common symptoms of NSAID hypersensitivity in both cohorts (89% vs. 77%, Swedish vs. Italian, respectively). Of cutaneous symptoms, flushing was the most common (26%), followed by angioedema (21%) and pruritus (21%). Urticaria and exanthema each occurred in 16% of patients. Respiratory symptoms occurred in 33% vs. 31%, cardiovascular symptoms in 22% vs. 35%, and gastrointestinal symptoms 6% vs. 15% respectively.

The frequency of NSAID-induced anaphylaxis was comparable in both cohorts, as anaphylaxis accounted for almost a quarter of overall NSAID-induced hypersensitivity reactions. Among patients with NSAID-hypersensitivity, 22% in the Swedish cohort and 27% in the Italian cohort reacted with anaphylaxis (Figure 1). Table 3 shows the clinical characteristics of patients who reacted with anaphylaxis (four from the Swedish cohort and seven from the Italian). In 8 of 11 patients, anaphylaxis was severe, affecting the cardiovascular system, with five patients presenting with syncope or loss of consciousness (Table 3).

**Table 3:** Clinical characteristics of patients who had a history of NSAID-induced anaphylactic reactions.

	**Diagnosis**	**Baseline tryptase**	**Total IgE**	**NSAID trigger**	**Clinical symptoms**
Patient nr 1	ISM	13 ng/ml	43 kU/L	Diclofenac	RESP, cutaneous (angioedema)
Patient nr 2	ISM	34 ng/ml	8 kU/L	Diclofenac	Cutaneous, CARDV, GI
Patient nr 3	ISM	11 ng/ml	22 kU/L	Diclofenac	Cutaneous, SYNC
Patient nr 4	MMAS	21 ng/ml	96 kU/L	Uncertain (ibuprofen and paracetamol in combination)	Cutaneous, RESP, SYNC
Patient nr 5	ISM	30 ng/ml	n/a	Nimesulide	Dyspnea, loss of consciousness
Patient nr 6	ISM	21 ng/ml	n/a	Nimesulide	Pruritus in hand palms and feet, urticaria, dyspnea, SYNC
Patient nr 7	MMAS	14 ng/ml	n/a	Ibuprofen	Dyspnea, tachycardia, hypotension, vertigo, SYNC
Patient nr 8	ISM	25 ng/ml	n/a	Nimesulide	Angioedema, dyspnea, hypotension
Patient nr 9	ISM	20 ng/ml	n/a	Diclofenac	Nausea, vomiting, diarrhea, fatigue, dyspnea
Patient nr 10	MMAS	56 ng/ml	n/a	Ibuprofen	Urticaria, hypotension
Patient nr 11	MMAS	19 ng/ml	50 kU/L	Diclofenac	Dyspnea, hypotension, SYNC, urticaria

Abbreviations: NSAID, non-steroidal anti-inflammatory drug; ISM, indolent systemic mastocytosis; MMAS, monoclonal mast cell activation syndrome; SYNC, syncope; CARDV, cardiovascular; RESP, respiratory; GI, gastrointestinal.

### Culprit NSAIDs

In the Swedish cohort, diclofenac was the most frequent elicitor of an NSAID-hypersensitivity reaction and accounted for 44% (8 of 18 patients), followed by ibuprofen 33% (6 of 18 patients). Furthermore, diclofenac was the culprit drug in 3 out of 4 patients (75%) who experienced anaphylaxis. In the fourth anaphylaxis patient, we were unable to identify a culprit agent as the patient had used ibuprofen and paracetamol in combination. In the Italian cohort, 20 of 26 patients reacted only once, 5 patients reacted twice, and one patient reacted thrice. Acetylsalicylic acid (ASA) was the most suspected elicitor and accounted for 30% of 33 reactions in 26 patients. However, nimesulide, which is not licensed for use in Sweden but is widely used in Italy, was the most common trigger of anaphylaxis, 43% (in three of seven cases). In remaining anaphylaxis cases, the culprits were diclofenac and ibuprofen, each accounting for the two cases (Table 3).

Overall, 34 patients underwent oral provocation tests to NSAIDs (Supplementary Table S1). Across the Italian cohort, 32 patients underwent a DPT using nimesulide. Of those, only eight had previously reacted to NSAIDs and DPTs were performed with an alternative NSAID to confirm a favorable tolerability. All eight patients tolerated nimesulide well. Two patients in the Swedish cohort underwent a DPT with ibuprofen, as both had previously had a mild reaction to this drug. Both tolerated it well.

### Risk factors of NSAID hypersensitivity in the Swedish cohort

Furthermore, we investigated potential risk factors for developing NSAID reactions in the Swedish cohort only, as standardized allergy work-up was not routinely performed in patients from the Italian cohort.

There were no differences in age, gender, bone-marrow findings, atopic status/diseases and sBT levels between mastocytosis patients who reacted to NSAIDs and those who were NSAID-tolerant (Table 4). In the MMAS patient group, however, total IgE levels were significantly increased among MMAS patients who reacted to NSAIDs compared to those who did not react (*p* = 0.004). No other statistically significant risk factors were identified.

**Table 4:** Comparison of patients with or without NSAID hypersensitivity in the Swedish cohort.

** **	Mastocytosis without NSAID hypersensitivity reaction (*n* = 156)	Mastocytosis with NSAID hypersensitivity reaction (*n* = 13)	*p*-value**^a^**	MMAS without NSAID hypersensitivity reaction (*n* = 28)	MMAS with NSAID hypersensitivity reaction (*n* = 5)	*p*-value^a^
**Age at diagnosis, median (range)**	52 (18–83)	50 (22–66)	0.503	51.5 (28–74)	55 (36–66)	0.706
**Male gender, *n* (%)**	72 (46.2)	5 (38.5)	0.774	15 (53.6)	1 (20.0)	0.335
**Presence of skin engagement, *n* (%)**	101 (64.7)	9 (69.2)	1.000	ND	ND	ND
**Presence of atopy, *n* (%)**	46 (29.5) (1 NA)	1 (7.7) (1 NA)	0.182	10 (35.7)	1 (20.0) (1 NA)	1.000
**Presence of asthma and/or rhinitis, *n* (%)**	40 (25.6)	2 (15.4) (2 NA)	0.732	12 (42.9)	2 (40.0) (1 NA)	1.000
**Total IgE levels (kU/L), median (range)**	14 (1–1600) (2 NA)	11.5 (4–280) (1 NA)	0.616	25 (6–1100)	170 (82–250)	**0**.**004**
**Baseline tryptase levels (ng/ml), median (range)**	29 (3–650)	34 (8–530)	0.981	9.0 (3–23)	17 (4–21)	0.191
**Occurrence of any anaphylaxis, *n* (%)**	72 (46.2)	6 (46.2)	1.000	20 (71.4)	3 (60.0)	0.627

Abbreviations: NA = not analysed. NSAID, non-steroidal anti-inflammatory drug; MMAS, monoclonal mast cell activation syndrome.

^a^ *p*-values were calculated using fisher's exact test.

^b^ *p*-values were calculated using a 2-tailed Mann-Whitney U-test; bold indicates statistical significance (*p* < 0.05).

We further analysed patients who had hypersensitivity to NSAIDs and compared mastocytosis with MMAS patients who reacted to NSAIDs (Table 5). Total IgE-levels were significantly higher in MMAS patients compared to mastocytosis patients (*p* < 0.01), although age, gender, presence of atopy, asthma/rhinitis or sBT levels did not significantly differ between groups (Table 5).

**Table 5:** Comparison between patients with mastocytosis and monoclonal mast cell activation syndrome who reacted with NSAIDs in the Swedish cohort.

	Mastocytosis with NSAID hypersensitivity reaction (*n* = 13)	MMAS with NSAID hypersensitivity reaction (*n* = 5)	*p*-value^a^
**Age at diagnosis, median (range)**	**50 (22–66)**	**55 (36–66)**	**0** **.** **430** **^b^**
**Male gender, *n* (%)**	**5 (38.5)**	**1 (20.0)**	**0** **.** **615** **^a^**
**Presence of atopy, *n* (%)**	**1 (7.7) (1 NA)**	**1 (20.0) (1 NA)**	**0** **.** **450** **^a^**
**Presence of asthma and/or rhinitis, *n* (%)**	**2 (15.4)**	**2 (40.0) (1 NA)**	**0** **.** **516** **^a^**
**Total IgE levels (kU/l), median (range)**	**11.5 (4–280) (1 NA)**	**170 (82–250)**	**<0** **.** **01** **^b^**
**Baseline tryptase levels (ng/ml), median (range)**	**34 (8–530)**	**17 (4–21)**	**0** **.** **217** **^b^**

Abbreviations: NSAID, non-steroidal anti-inflammatory drug; MMAS, monoclonal mast cell activation syndrome NA = not analysed.

^a^ *p*-values were calculated using Fisher's exact test;.

^b^ *p*-values were calculated using a 2-tailed Mann-Whitney U-test; Bold indicates statistical significance (*p* < 0.05).

In the total study cohort, females were predominantly represented among patients with anaphylaxis (64%, 7/11).

## Discussion

In this study, we investigated prevalence and clinical features of hypersensitivity reactions to NSAIDs in two independent large cohorts of patients with clonal MCD, mostly mastocytosis, and demonstrated an increased risk compared to the general population. To the best of our knowledge, with reference to NSAID hypersensitivity, this is the largest studied cohort in adult mastocytosis patients so far.

We determined an overall prevalence of hypersensitivity to NSAIDs as 11.3% in the total study cohort. This is a 4- to 5-fold increased risk compared to the general population, where a prevalence of 0.6 to 2.5% has been reported (16). However, this is similar to adult asthmatic patients, where the prevalence NSAID hypersensitivity ranges from 4.3 to 11% (23, 33). Moreover, among patients with asthma and nasal polyps, the prevalence may reach 25.6% (33), and may be as high as 27%–35% in patients with chronic urticaria (22, 34). Hence, considering these conditions, the prevalence of NSAID-hypersensitivity among mastocytosis patients does not appear to be remarkably high. Nevertheless, an important conclusion from our study is that the frequency of NSAID hypersensitivity and anaphylaxis is higher in MMAS than in mastocytosis. Although the statistical significance will depend on sample size and frequency of events, the trend seems quite clear. Furthermore, when comparing our results to previous studies in mastocytosis patients, our study is consistent with the Spanish study showing a 13% frequency of hypersensitivity to NSAIDs among adult patients with mastocytosis (25). Conversely, we determined an approximately 3-fold increase compared to a Dutch study reporting a prevalence of 4.1% (24). Interestingly, when authors performed oral challenges in 50 of these patients, the actual prevalence was only 2%; i.e., even lower (24). Similarly, this may also be the case in our study since no patients who underwent DPTs reacted.

Most hypersensitivity reactions to NSAIDs were mild and limited to the skin in our study, as 89% of hypersensitivity patients presented with cutaneous lesions, with flushing being the most common reaction pattern (26%). The second most common symptoms were respiratory symptoms with an overall prevalence of approximately 3.6%. Another study examining the prevalence of NSAID-induced respiratory symptoms in a population across 15 centers in 22 European countries found a slightly lower overall prevalence of 1.94% (35).

NSAIDs have been reported to be the major (36) or second group of drugs (19) responsible for drug-induced anaphylaxis. In this study, around 25% of patients with NSAID-hypersensitivity were deemed to have had anaphylactic reactions. This is consistent with a study evaluating NSAID-hypersensitivity in the general population, where anaphylaxis accounted for 23% of all hypersensitivity reactions (20). Another study, however, examining data for 1,137 patients with NSAID-hypersensitivity in an allergy clinic reported 11% anaphylaxis, which is lower than ours (23). We found an overall prevalence of NSAID-induced anaphylaxis in 2.8% of our total study cohort. When we compare this to the previous studies of mastocytosis patients, this is three times lower than the Spanish study, which reported a 9% overall prevalence of anaphylaxis (accounting for 66% of overall hypersensitivity reactions to NSAIDs) among mastocytosis patients (25). Nevertheless, this is higher than the Dutch study reporting 1.5% overall anaphylaxis prevalence (corresponding to 38% of overall NSAID-hypersensitivity reactions) (24). Several factors may explain such discrepancies between different populations. First, anaphylaxis definition, such as interpreting anaphylaxis criteria, and methods may differ between case series. Second, genetic background, which is associated with higher risk of developing reactions to a specific drug, varies from one population to another. Finally, drugs are used differently across regions depending upon availability. Interestingly however, if we compare the risk of NSAID-induced anaphylaxis to risk of venom-induced anaphylaxis (VIA) in mastocytosis patients, there are huge differences: among patients with anaphylaxis in this cohort, 28% reacted to insect venoms. Thus, the overall risk for NSAID-induced anaphylaxis is still 10-fold lower compared to VIA. Nevertheless, there is almost the same frequency of food-induced anaphylaxis in mastocytosis, as an overall prevalence of 2.5% was reported (37).

There were some differences between cohorts regarding culprit NSAIDs. Diclofenac was the most suspected NSAID drug causing 44% of all reported reactions and 75% of anaphylaxis in the Swedish cohort. This is in line with a study from Austria (38). Nevertheless, ASA (30%) was the most suspected elicitor in the Italian cohort. Similarly, nimesulide accounted for 43% of anaphylactic reactions, followed by diclofenac (29%) and ibuprofen (29%). Interestingly, it has been debated whether patterns of consumption of NSAIDs may be reflected in the prevalence of hypersensitivity reactions, as availability of different NSAIDs differ in different countries. A retrospective study involving 659 patients with NSAID-hypersensitivity reported that culprit NSAIDs changed over three decades (39). However, this rule has not always been followed. For instance, naproxen is the most frequently prescribed NSAID in Sweden corresponding to 39% of total consumption, followed by diclofenac (19%) and ibuprofen (15%) (https://sdb.socialstyrelsen.se/if_lak/val.aspx). Interestingly, however, most reactions in the Swedish cohort were caused by diclofenac. In contrast, no patients in the Swedish cohort had a history of reactions to nimesulide and metamizole, since these drugs are not available in the Swedish market. Hence, it is not always feasible to compare culprits of NSAIDs due to regional and cultural differences. Paracetamol is available over-the-counter and widely used even in patients with history of NSAID-hypersensitivity in Sweden. In this study, all patients with previous adverse reactions triggered by NSAIDs, in both cohorts, tolerated paracetamol.

In the general population, the influence of female gender or atopic predisposition have been shown as risk factors in some reports (40, 41), but not others (42). In the current study, total IgE levels were significantly higher in MMAS patients with NSAID hypersensitivity compared to NSAID-tolerant patients (*p* = 0.004). Nevertheless, consistent with a previous report (21), we could not detect any significant risk factors related to gender, atopic predisposition, sBT levels, history of anaphylaxis or comorbidity with asthma and/or rhinitis. However, females were more at risk in both cohorts with an overall frequency of 64% among patients with NSAID-induced anaphylaxis.

The main strength of our study is that it includes data from two independent large cohorts of well-characterized mastocytosis patients. Centers were located in different regions of Europe and also practice patterns and processes of patient care somewhat differ between them, thus increasing the generalizability of our findings and enhancing our validation of findings from previous studies. However, we also recognize that our study has certain limitations. Firstly, its retrospective nature is an inherent weakness. Secondly, there may be recall bias, as the diagnosis of NSAID hypersensitivity was based on a reported history and further assessment of an allergist. Cases with unreliable history were excluded, as DPTs were not systematically performed (only 34 patients underwent a DPT, most of them with the alternative NSAIDs). This might have altered the actual prevalence of hypersensitivity to NSAIDs. An additional limitation is that, although most patients reported that reactions occurred shortly after drug intake, not all recalled the exact timing.

In conclusion, although the prevalence of NSAID reactions appear to be more common in patients with clonal MCD, severe reactions, such as NSAID-induced anaphylaxis are still infrequent. Additionally, all anaphylactic reactions occurred prior to a diagnosis of mastocytosis being obtained. Because there is presently limited data on NSAID-hypersensitivity in patients with clonal MCD, our data may provide important information about frequency, severity and safety of NSAIDs in this group of patients. As these drugs are available over-the-counter in most countries, our results support the proposal that patients with a known tolerance to NSAIDs can continue using these medications without caution, whereas those with a prior reaction to NSAIDs should undergo thorough allergy work-up, including drug challenges.

## Data Availability

The data are not publicly available due to privacy or ethical restrictions. The raw data supporting the findings of this study will be made available on request from the authors.
