# Supplementary material for: Non-steroidal anti-inflammatory drug-induced anaphylaxis infrequent in 388 patients with mastocytosis: A two-center retrospective cohort study
Source: Front Allergy. 2022 Dec 5;3:1071807. doi: 10.3389/falgy.2022.1071807 (PMC9760711; doi:10.3389/falgy.2022.1071807)
Supplement: Supplementary file 1 [file Table1.pdf]

Supplementary Table S1: Clinical characteristics of mastocytosis patients (n=34) who underwent drug provocation tests

NSAID Culprit	Diagnosis	Age at Diagnosis, Gender	Reaction pattern	DPT	Reaction
1. **None**	ISM	45, F	None. Reluctant to use NSAID due to anxiety	Nimesulide	None
2. **None**	ISM	59, M	None. Reluctant to use NSAID due to anxiety	Nimesulide	None
3. **None**	ISM	52, F	None. Reluctant to use NSAID due to anxiety	Nimesulide	None
4. **None**	ISM	42, F	None. Reluctant to use NSAID due to anxiety	Nimesulide	None
5. **None**	MMAS	58, F	None. Reluctant to use NSAID due to anxiety	Nimesulide	None
6. **Naproxen**	ISM	50, M	Urticaria	Nimesulide	None
7. **None**	MMAS	62, M	None. Reluctant to use NSAID due to anxiety	Nimesulide	None
8. **None**	ISM	40, F	None. Reluctant to use NSAID due to anxiety	Nimesulide	None
9. **None**	ISM	66, F	None. Reluctant to use NSAID due to anxiety	Nimesulide	None
10. **None**	ASM	47, F	None. Reluctant to use NSAID due to anxiety	Nimesulide	None
11. **None**	MMAS	40, F	None. Reluctant to use NSAID due to anxiety	Nimesulide	None
12. **None**	MMAS	65, F	None. Reluctant to use NSAID due to anxiety	Nimesulide	None
13. **None**	ISM	22, F	None. Reluctant to use NSAID due to anxiety	Nimesulide	None
14. **None**	ISM	25, M	None. Reluctant to use NSAID due to anxiety	Nimesulide	None
15. **None**	ISM	37, F	None. Reluctant to use NSAID due to anxiety	Nimesulide	None
16. **None**	ISM	37, M	None. Reluctant to use NSAID due to anxiety	Nimesulide	None
17. **None**	ISM	63, F	None. Reluctant to use NSAID due to anxiety	Nimesulide	None
18. **None**	ISM	56, M	None. Reluctant to use NSAID due to anxiety	Nimesulide	None
19. **None**	ISM	61, F	None. Reluctant to use NSAID due to anxiety	Nimesulide	None
20. **None**	ISM	41, F	None. Reluctant to use NSAID due to anxiety	Nimesulide	None
21. **None**	ISM	67, F	None. Reluctant to use NSAID due to anxiety	Nimesulide	None
22. **None**	ISM	70, F	None. Reluctant to use NSAID due to anxiety	Nimesulide	None
23. **None**	ISM	55, F	None. Reluctant to use NSAID due to anxiety	Nimesulide	None

**24. None**	ISM	35, F	None. Reluctant to use NSAID due to anxiety	Nimesulide	None
**25. None**	ISM	49, M	None. Reluctant to use NSAID due to anxiety	Nimesulide	None
**26. ASA**	ISM	56, F	Urticaria	Nimesulide	None
**27. ASA**	MMAS	48, F	Urticaria, angioedema	Nimesulide	None
**28. ASA**	SSM	80, F	Urticaria	Nimesulide	None
**29. ASA**	ISM	49, F	Palpitation, vertigo	Nimesulide	None
**30. Diclofenac**	MMAS	56, M	Anaphylaxis (dyspnea, urticaria, hypotension)	Nimesulide	None
**31. Ketoprofen**	MIS	58, F	Angioedema	Nimesulide	None
**32. ASA**	ISM-AHN	59, M	Urticaria, flushing	Nimesulide	None
**33. Ibuprofen**	ISM	51, M	Pruritus, flushing	Ibuprofen	None
**34. Ibuprofen**	ISM	53, M	Exantema	Ibuprofen	None

Abbreviations: NSAID, non-steroidal anti-inflammatory drug; DPT, drug provocation test; ISM, indolent systemic mastocytosis; ISM-AHN, indolent systemic mastocytosis with an associated hematologic neoplasm; MIS, mastocytosis in the skin; MMAS, monoclonal mast cell activation syndrome; F, female; M, male.
